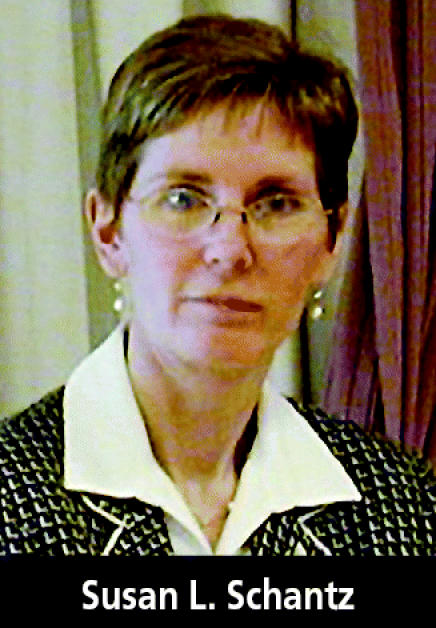# Editorial: Lakes in Crisis

**DOI:** 10.1289/ehp.113-a148

**Published:** 2005-03

**Authors:** Susan L. Schantz

**Affiliations:** University of Illinois at Urbana–Champaign, Urbana, Illinois, E-mail: schantz@uiuc.edu

As Scott Fields points out in his Focus article (Fields 2005), the Great Lakes are one of the most important water resources on Earth. Together the five lakes contain around 20% of the surface freshwater on the planet. The Great Lakes basin is home to close to 40 million people in the United States and Canada, and it is no accident that some of the largest and most heavily industrialized cities in North America are located there. During the industrial expansion the lakes provided a ready transportation system for delivering raw materials to factories and products to markets. They also provided a seemingly endless supply of water for industrial processes and acted as a convenient sewer for industrial waste. Ironically the same lakes that have been so heavily used and abused by industry support the largest fresh-water fishery in the world, and both commercial and sport fishing are critical to the economy of the region.

Over the last century the once pristine waters of the five lakes—Superior, Michigan, Huron, Erie, and Ontario—have become contaminated with a virtual soup of toxic chemicals. Most worrisome among these are chlorinated organic compounds that persist for long periods of time in the sediment of the lakes, enter the food chain, and bioaccumulate as they move from phytoplankton to zooplankton, to forage fish, and finally to predatory fish such as lake trout and salmon. Chief among these are DDE (dichlorodiphenyldichloroethylene), a breakdown product of the once widely used herbicide DDT (dichlorodiphenyltrichloroethane), and polychlorinated biphenyls (PCBs), once used as dielectric fluids in capacitors and transformers. Decades of research has revealed that these endocrine-disrupting chemicals can cause a host of health problems in wildlife and humans. The good news is that concentrations of PCBs and DDE in Great Lakes fish peaked in the 1970s and are declining. The bad news is that, because of their extreme stability, these chemicals will continue to be present in fish at levels that could represent a health risk for decades to come.

Despite our experiences with DDE and PCBs, we seem destined to repeat our earlier mistakes. Recently, a new class of contaminants—the polybrominated diphenyl ethers (PBDEs)—has exploded onto the scene. PBDEs, which are chemically similar to PCBs, are used as flame retardants in a myriad of products. Over the last 10–15 years the concentrations of PBDEs in Great Lakes fish have been increasing exponentially, and this trend shows no signs of abating. This buildup is of particular concern because of the potential for additive effects from combined exposure to PBDEs and their close chemical cousins, the PCBs.

Pollutants are by no means the only crisis facing the Great Lakes in the 21st century. Non-native species have been stealthily making their way into the lakes for many years, wreaking havoc on a fragile and delicately balanced ecosystem. An excellent series of articles, which was published recently in the *Milwaukee Journal Sentinel* (Egan 2004a, 2004b, 2004c), details Lake Michigan’s evolution over the last 50 years as these interlopers have arrived and taken up residence.

The story began with the arrival of the sea lamprey in the 1950s. At that time the top predatory fish in the lake—the lake trout—was already in peril due to overfishing and habitat degradation. The sea lamprey quickly wiped out what was left of the trout population. With no predatory fish at the top of the food chain, another invasive species—the common alewife—began to flourish. By the1960s, alewives had become so plentiful that they began dying off by the billions, polluting beaches with tons of rotting fish.

In the mid-1960s, the discovery of a poison that could control the sea lamprey made it possible to reintroduce large, predatory fish to the lake. However, instead of focusing on restoring the native lake trout, at this critical juncture the decision was made to introduce salmon—another foreign species—to the lake. The yearly stocking of Lake Michigan with hatchery-born salmon solved the alewife problem and created a sport fishing paradise. However, what goes around comes around. Eventually, the overstocking of salmon led to a precipitous decline in the alewife population, and starving salmon began washing up on the same beaches that had been littered with alewife several decades earlier. In an attempt to protect the now-prized salmon population, officials acted to restore the once reviled alewife population.

Unfortunately, the story does not end there. As all of this was unfolding, a tiny creature slipped almost unnoticed into the lake, quickly multiplied, and began eating away at the very foundation of the Lake Michigan food web. The zebra mussel had arrived. It was not long before the impact of these tiny crustaceans was reverberating throughout the Great Lakes basin. In Lake Michigan, food sources for the now-important alewife as well as for the whitefish and perch, two native fish species that are still hanging on in the lake, began to disappear.

Currently the salmon fishing in Lake Michigan is great because the alewife population has again plummeted and the fish are so hungry they will bite on just about anything. Meanwhile, perch fry are dying off because they cannot get enough to eat, and hungry white-fish are trying to consume the zebra mussels, which are tearing their insides apart. The lake is once again teetering on the brink of disaster.

We have reached a critical juncture in the life of the Great Lakes. These precious waters and the wildlife they support are one of the most important natural resources on planet. If we do not act now to develop a far-reaching plan for restoration of the lakes, we will soon arrive at the point where there is nothing left to restore. A bill currently pending in the U.S. Congress (Senate bill 1398) directs the states to work with the federal government to restore the Great Lakes. The bill would provide $6 billion over 5 years to set the restoration process in motion. All of us should be doing our part to ensure that this bill moves through Congress rapidly and is signed into law.

The road to restoration will not be easy. To be successful it will require bold leadership, innovative thinking, and the courage to implement politically and economically unpopular decisions. It will require adequate funding for research to find ways to control the invasive species that have already populated the lakes, tough regulations on shipping and strict enforcement of those regulations to prevent further colonization of the lakes, and a move away from stocking the lakes with exotic species such as salmon and rainbow trout that currently contribute significantly to the regional economy through the recreational fishing industry. It will also require quick and decisive action to regulate emerging contaminants such as PBDEs before they accumulate to the point that they become a health risk to wildlife and humans living in and around the lakes. The Council of Great Lakes Governors should take a leadership role in moving the restoration of this precious resource forward.

## Figures and Tables

**Figure:**